# Integrative Analysis of mRNA Expression and Half-Life Data Reveals *Trans*-Acting Genetic Variants Associated with Increased Expression of Stable Transcripts

**DOI:** 10.1371/journal.pone.0079627

**Published:** 2013-11-18

**Authors:** Thong T. Nguyen, Cathal Seoighe

**Affiliations:** 1 School of Mathematics, Statistics & Applied Mathematics, National University of Ireland, Galway, Ireland; 2 Institute of Infectious Disease and Molecular Medicine, University of Cape Town, Anzio Road, Observatory, Cape Town, South Africa; German Cancer Research Center, Germany

## Abstract

Genetic variation in gene expression makes an important contribution to phenotypic variation and susceptibility to disease. Recently, a subset of *cis*-acting expression quantitative loci (eQTLs) has been found to result from polymorphisms that affect RNA stability.

Here we carried out a search for *trans*-acting variants that influence RNA stability. We first demonstrate that differences in the activity of *trans*-acting factors that stabilize RNA can be detected by comparing the expression levels of long-lived (stable) and short-lived (unstable) transcripts in high-throughput gene expression experiments. Using gene expression microarray data generated from eight HapMap3 populations, we calculated the relative expression ranks of long-lived transcripts versus short-lived transcripts in each sample. Treating this as a quantitative trait, we applied genome-wide association and identified a single nucleotide polymorphism (SNP), rs6137010, on chromosome 20p13 with which it is strongly associated in two Asian populations (*p = *4×10^−10^ in CHB – Han Chinese from Beijing; *p* = 1×10^−4^ in JPT – Japanese from Tokyo). This SNP is a *cis*-eQTL for *SNRPB* in CHB and JPT but not in the other six HapMap3 populations. *SNRPB* is a core component of the spliceosome, and has previously been shown to affect the expression of many RNA processing factors.

We propose that a *cis*-eQTL of *SNRPB* may be directly responsible for inter-individual variation in relative expression of long-lived versus short-lived transcript in Asian populations. In support of this hypothesis, knockdown of *SNRPB* results in a significant reduction in the relative expression of long-lived versus short-lived transcripts. Samples with higher relative expression of long-lived transcripts also had higher relative expression of coding compared to non-coding RNA and of RNA from housekeeping compared to non-housekeeping genes, due to the lower decay rates of coding RNAs, particularly those that perform housekeeping functions, compared to non-coding RNAs.

## Introduction

RNA stability plays a major role in gene expression regulation in virtually all organisms, from bacteria to mammals [1], [2], [3]. Indeed, steady-state gene expression levels represent the equilibrium of two opposing biological processes: RNA transcription and RNA decay. Changes in gene expression levels can result from alteration in either of these processes [1], [4]. Recent studies have investigated RNA stability using high-throughput techniques in diverse organisms, from yeast [5], [6] to *Arabidopsis* [7], mouse [8], [9], [10], and human [10], [11], [12], [13], [14], and for both coding and non-coding RNAs [9], [15]. Several of these studies have reported strong correlations between RNA stability and steady-state gene expression levels. In addition, RNA stability has been shown to be related to physiological function [8], [12]. For example, genes encoding proteins involved in housekeeping functions tend to have stable mRNAs [10], [15]. The modulation of RNA stability can, in turn, have a major impact on cellular processes, including proliferation, differentiation, and adaptation to environmental stimuli [1], [2], [3]. Dysregulation of RNA stability has been linked to several human diseases, such as chronic inflammation [16], cardiovascular disease and cancer [17], [18], [19].

The regulation of RNA stability is achieved through interactions between *trans*-acting RNA-binding proteins and *cis*-acting elements within RNAs [20], [21]. Among RNA-binding proteins, heterogeneous nuclear ribonucleoproteins (hnRNPs) are key factors that regulate major steps of gene expression, including pre-mRNA processing, RNA stability, and translation [22], [23], [24]. For example, *HNRNPA2B1*, a member of the hnRNP family, was found to stabilize a large number of target transcripts carrying a conserved structural RNA element in the 3′ untranslated regions [13]. Knockdown of *HNRNPA2B1* resulted in a remarkable increase in the relative decay rate of the target transcripts and, consequently, a significant decrease in their expression levels [13]. The contribution of RNA decay to gene expression levels was also investigated in a recent study where a subset of *cis*-acting expression quantitative loci (*cis*-eQTLs) was found to be a consequence of variation in decay rates [25]. A moderate number of genetic variants were found to significantly associate with inter-individual variation in both gene expression and RNA decay, for which variation in RNA decay could explain the association with gene expression level [25]. Despite increased appreciation of the role of RNA stabilization in determining gene expression levels there has been no investigation of *trans*-acting genetic variants that affect the stabilization of RNA.

Here we investigate factors that affect RNA stability in *trans*. We first show that perturbation of RNA stabilization factors that affect multiple genes can be inferred from gene expression data. Given a dataset of RNA decay rates and expression levels, we define the RNA stability score (RS-score), based on the expression of long-lived transcripts relative to short-lived transcripts. Knocking down *HNRNPA2B1*, which has been shown to be involved in stabilization of a large proportion of RNAs [13], leads to a significant reduction in the RS-score. Using gene expression microarray data generated from eight HapMap3 populations [26], we identified a SNP, rs6137010, on chromosome 20p13 that is strongly associated with the RS-score in Asian populations. This SNP is a *cis*-eQTL of *SNRPB*, a gene that encodes a core component of the spliceosome and has been shown to modulate the expression of many RNA processing factors [27]. The C allele of rs6137010 is associated both with higher expression of *SNRPB* and higher RS-score. Knockdown of *SNRPB* results in a significant decrease in the RS-score, suggesting that the *cis*-eQTL for *SNRPB* is responsible for the observed genetic variation in RS-score in Asian populations.

## Results and Discussion

### Perturbation of RNA stabilization is detectable from gene expression data

We hypothesized that changes in the activity of *trans*-acting factors that are involved in stabilizing multiple RNAs could be detectable by analyzing gene expression profiles. To test this hypothesis we obtained gene expression data from a published study in which the heterogeneous ribonucleoprotein, *HNRNPA2B1*, was knocked down [13]. In the original study this gene was shown to play a role in the stabilization of RNAs containing an abundant structural motif and RNAs containing this motif were downregulated in the knockdown samples compared to controls [13]. However, even in the absence of knowledge of the specific *trans*-acting factor and target RNAs involved it is possible to infer the effects of the knockdown on RNA stability. This is because stable, long-lived transcripts are enriched among the genes that are targeted by *HNRNPA2B1* [13].

We divided genes into two groups by using RNA decay rate data from Goodarzi *et al*. [13]. The first group contains genes expressing long-lived RNAs (decay rate lower than the mean across genes) and the second group contains genes expressing short-lived RNAs (decay rate higher than the mean). We then defined the RS-score for a sample as the difference in the expression rank between these two groups of genes in the sample (see Methods for more details). A higher RS-score implies relatively higher expression levels of long-lived or stable RNAs. A similar idea has previously been used to infer the impact of miRNA regulation on target genes using gene expression data [28]. The regulatory effect score (RE-score) of a miRNA was defined as the difference in the mean expression rank between targets of the miRNA and non-targets. A higher RE-score indicates lower expression levels of target genes and, thereby, a stronger effect of the corresponding miRNA. Analogously, a higher RS-score implies that the long-lived RNAs that are more likely to be subject to stabilization by *trans*-acting factors are relatively more highly expressed in a sample.

**Figure 1:**
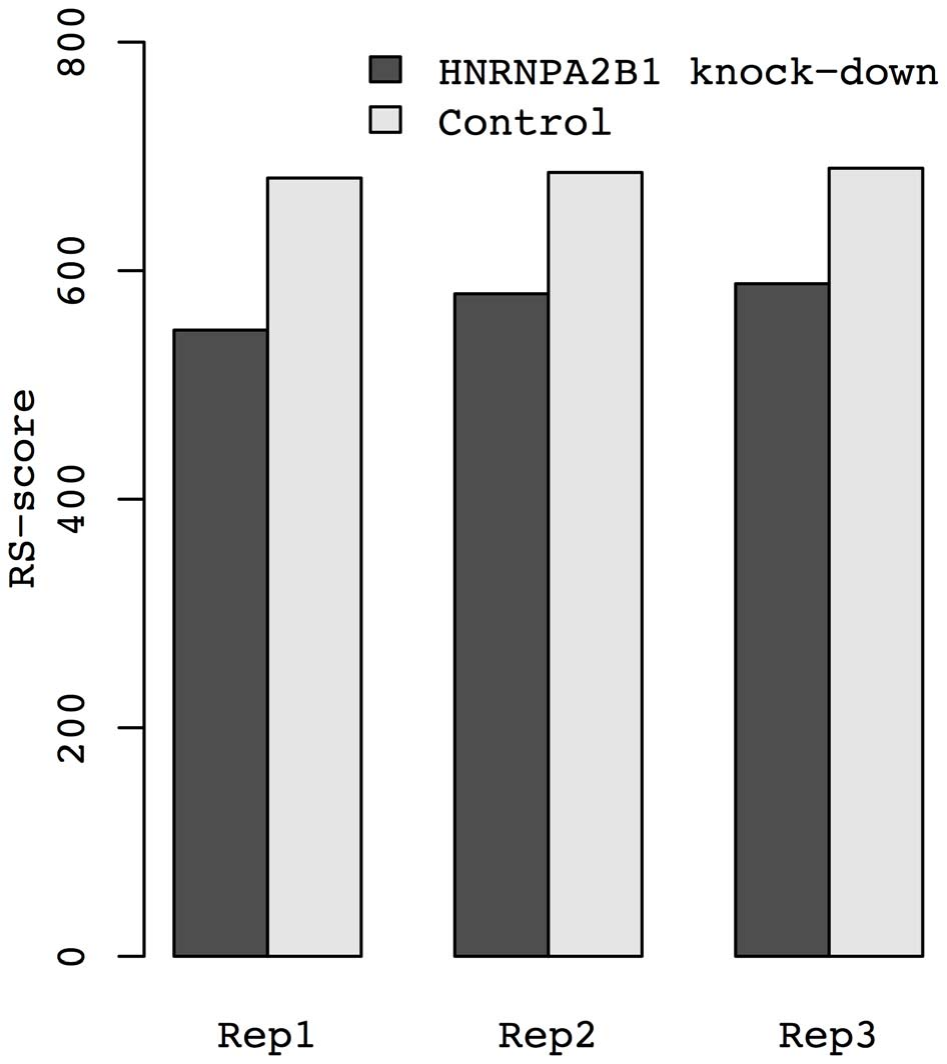
*HNRNPA2B1* knock-down results in reduced RS-score. RS-score was calculated for *HNRNPA2B1* knockdown samples and control samples separately in three independent replicates (Rep1, Rep2, and Rep3).

The RS-score of the *HNRNPA2B1* knockdown was significantly lower than RS-score of the control in three independent replicates (*p* = 3.7×10^−3^; paired *t* test) (Figure 1). This is consistent with expectations because *HNRNPA2B1* is one of the heterogeneous nuclear ribonucleoproteins that influence pre-mRNA processing and other aspects of RNA metabolism and transport. More importantly, *HNRNPA2B1* is involved in stabilizing a large number of genes, particularly genes expressing long-lived RNAs, by binding to a structural RNA motif of target genes [13]. *HNRNPA2B1* knockdown caused a significant reduction in the expression levels of long-lived RNAs (Figure S1), resulting in lower RS-scores in the knockdown samples. These observations suggest that gene expression levels can be used to infer the effects of *trans*-acting factors that are involved in stabilizing large numbers of genes.

### The genetics of *trans*-acting factors that affect RNA stability

We obtained gene expression data generated from lymphoblastoid cell lines of 726 individuals in eight HapMap3 populations [26] (Table S1). Using the half-life data from HeLa cells [15], we calculated the RS-score for each of these individuals (see Methods). Interestingly, the RS-score was well correlated with the expression level of *HNRNPA2B1* in most of the populations (Table S2), with the strongest correlation in CHB (Spearman *rho* = 0.48; p = 8.4×10^−6^). Because the experimental knock down of *HNRNPA2B1* results in a reduction in the RS-score, we hypothesized that *cis*-eQTLs affecting the expression level of *HNRNPA2B1* should also be associated with RS-score. This is the case for four *cis*-eQTLs of this gene in two of the HapMap3 populations (Table S3).

**Figure 2:**
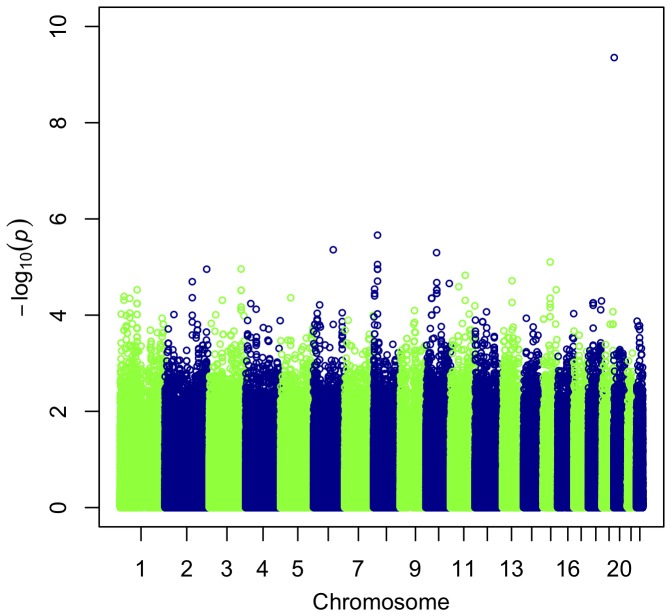
Manhattan plot for GWA with RS-score in CHB. The plot shows -log_10_ of P-values from tests of association between individual SNP markers and the RS-score. Successive chromosomes are shown in different colors.

To search more generally for genetic variants associated with the RS-score we used a genome-wide association study (GWAS) approach, treating the RS-score as a quantitative trait. We carried out additive tests of association between single nucleotide polymorphisms (SNPs) genotyped as part of the HapMap3 project and the RS-score in each population separately (see the Methods section for more details). We found one strong association between a SNP, rs6137010, on chromosome 20p13 and RS-score in the CHB population (*p* = 4.4×10^−10^; Figure 2). Interestingly, this association is replicated in the other Asian population – JPT (*p* = 1.2×10^−4^). We used a label permutation procedure to check the robustness of this result to failures in modelling assumptions (see Methods). The association between rs6137010 and RS-score in CHB was stronger than the best associations in each of 1,000 label permutations. Furthermore, the Bonferroni-adjusted p-value of this association is very significant (Bonferroni *p* = 5.9×10^−3^). Therefore, the association between rs6137010 and RS-score in CHB is robust, genome-wide significant, and replicated in a second population (JPT).

**Figure 3:**
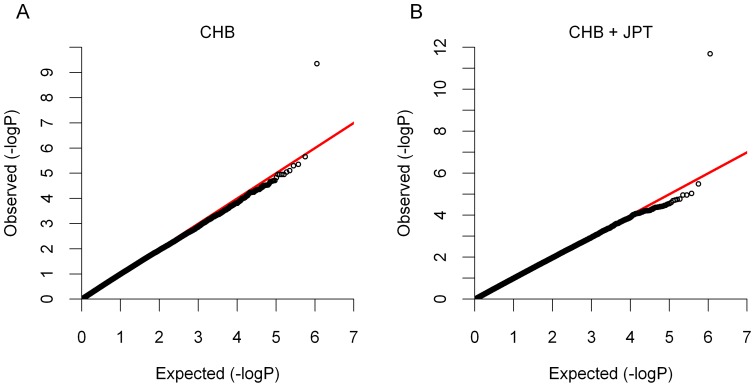
P-P plots of the association with RS-score in A) CHB and B) CHB+JPT. This figure compares the observed distribution of the –log_10_ P-values to the expected distribution, given that the P-values come from a uniform distribution in the interval zero to one (as expected under the null hypothesis). The Y-axis shows quantiles of the observed distribution and the X-axis shows the corresponding quantiles under the uniform distribution. The red line is used to compare the expected and observed values.

To increase the statistical power of the association tests, we combined individuals from different populations. Because different populations have different ancestries combining individuals from these populations can lead to spurious associations, resulting from structure in the combined population. To tackle this problem, we applied a principal components analysis (PCA) approach [29] (see Methods for more details) to model ancestry differences among all 726 individuals. In a scatter plot of the first and second principal components (Figure S2) three broad clusters are evident, consisting of the African populations, the Asian populations and CEU, MEX, GIH. Given these clusters, we considered four ways of combining populations: CHB+JPT (Asian populations), YRI+MKK+LWK (African populations), CEU+GIH+MEX, and finally all 8 populations (ALL). For each combination, we performed a principal components analysis and included the first five principal components as covariates in the GWAS regression models (see Methods). The SNP rs6137010 was strongly associated with the RS-score in CHB+JPT (*p* = 2.0×10^−12^; Figure S3). This association is also the best among 1000 permutations and is genome-wide significant (Bonferroni *p* = 2.7×10^−5^). In total, 6 genetic markers showed genome-wide significant association (Bonferroni *p*<0.05) but the association with rs6137010 in CHB+JPT was the strongest (Table 1). The P-P plots showed that the p-value of the association with the RS-score at rs6137010 is very different to other loci in the Asian populations (Figure 3). We found no evidence of population stratification in the GWAS tests of the Asian populations as their genomic inflation factors are less than 1.05 (Table S4). However, unsurprisingly there was evidence of population stratification in three combined populations: CEU+GIH+MEX, YRI+LWK+MKK and ALL (Table S4; Figure S4).

**Table 1:** Markers associated with the RS-score at Bonferroni *p*<0.05.

SNP	Location	Function	Associated gene	Population	P-value	Bonferroni
rs6137010	20:2038118	*cis*-eQTL	SNRPB	CHB+JPT	2.0×10^−12^	2.7×10^−5^
rs6137010	20:2038118	Intron	STK35	CHB+JPT	2.0×10^−12^	2.7×10^−5^
rs6137010	20:2038118	*cis*-eQTL	SIRPA	ALL	5.4×10^−11^	9.0×10^−4^
rs6137010	20:2038118	intron	STK35	ALL	5.4×10^−11^	9.0×10^−4^
rs11136253	8:145179783	*cis*-eQTL	ZNF707	ALL	1.5×10^−10^	2.5×10^−3^
rs11136253	8:145179783	coding-synon	OPLAH	ALL	1.5×10^−10^	2.5×10^−3^
rs6137010	20:2038118	*cis*-eQTL	SNRPB	CHB	4.4×10^−10^	5.9×10^−3^
rs6137010	20:2038118	Intron	STK35	CHB	4.4×10^−10^	5.9×10^−3^
rs4466324	7:85113458	unknown	None	ALL	6.0×10^−10^	1.1×10^−2^
rs17127419	11:122878168	unknown	HSPA8	ALL	9.8×10^−10^	1.6×10^−2^
rs12034707	1:178400832	*cis*-eQTL	TOR1AIP1	ALL	1.8×10^−9^	3.0×10^−2^
rs12034707	1:178400832	intron	QSOX1	ALL	1.8×10^−9^	3.0×10^−2^
rs10997765	10:69066422	intron	CTNNA3	ALL	1.9×10^−9^	3.3×10^−2^

To check the effect of the choice of half-life data on this result, we compared RS-scores calculated using half-life data from HeLa cells and RS-scores calculated using B-cell half-life data [14] and found that they were highly correlated in all populations (Spearman *rho* = 0.73±0.15). It has previously been reported that RNAs involved in housekeeping functions tend to have long half-life [10], [15]. As an alternative to using half-life data, which has the caveat that it may be cell type dependent, we calculated the RS-score by grouping genes based on whether they are housekeeping or not, using data from Chang *et al*. [30]. We found that the RS-score calculated by grouping the genes in this way was highly correlated with the RS-score based on the half-life in HeLa cells in all populations (Spearman *rho* = 0.70±0.09). Moreover, the RS-score (based on the housekeeping data) was significantly associated with rs6137010 in the combined CHB+JPT population (*p* = 7.1×10^−13^; Bonferroni *p* = 9.5×10^−6^). We also calculated an equivalent score by considering protein-coding versus non-coding genes. Non-coding genes have been found to have shorter half-life than protein-coding genes [9], [15]. This score was also highly correlated with the RS-score calculated from the half-life data and, again, significantly associated with rs6137010 in CHB+JPT (*p* = 6.2×10^−10^; Bonferroni *p* = 8.3×10^−3^). These two results are of interest, beyond providing an alternative way to group genes that is not dependent on RNA half-life data that may differ between cell types. They suggest that the proportion of the RNA pool corresponding to non-coding and tissue-specific genes is associated with rs6137010 in Asian populations.

### Searching for causal SNPs and causal genes

**Figure 4:**
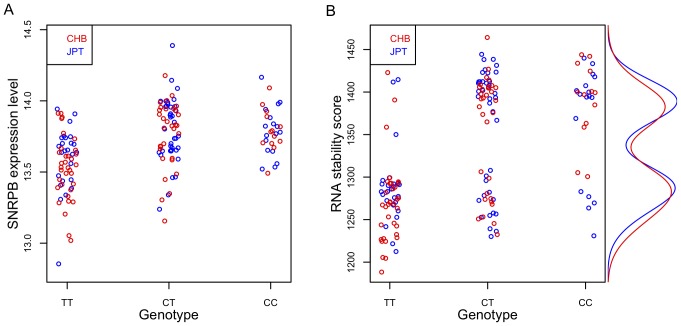
Stripcharts of *SNRPB* expression levels and the RS-score against the genotype of rs6137010 in CHB and JPT. A) *SNRPB* expression levels are significantly different among the three genotypes TT, CT and CC (*p* = 1.2×10^−5^ in CHB and *p* = 1.9×10^−3^ in JPT from one-way ANOVA). B) RS-scores are significantly different among the three genotypes (*p* = 4.3×10^−10^ in CHB and *p* = 7.0×10^−5^ in JPT from one-way ANOVA). The bimodal distributions of the RS-score in CHB and JPT are displayed in red and blue lines, respectively.

To search for causal SNPs that may explain the GWAS results we mapped each SNP that shows genome-wide significant association with the RS-score to a gene if the SNP is either within the gene or is a *cis*-eQTL (*cis*-expression Quantitative Trait Locus) of the gene using *cis*-eQTL data from Stranger *et al*. [26] (Table 1). We found that rs6137010, the SNP with the strongest GWAS signal, mapped close to the *SNRPB* gene, which is involved in RNA processing. *SNRPB* encodes part of the core small nuclear ribonucleoprotein particles (snRNPs) that are major components of the spliceosome complex. Although it is 352 kb downstream, rs6137010 is significantly associated with the expression level of *SNRPB* in both CHB (*rho* = 0.50; *p* = 2.3×10^−6^) and JPT (*rho* = 0.32; *p* = 3.7×10^−3^), but not significantly associated with *SNRPB* expression in any of the other populations studied. The association between rs6137010 and *SNRPB* is strongest among all genes within 1 Mb-window centered on the SNP. Furthermore, the SNP is within an enhancer region as evidenced from whole-genome chromatin state segmentation data [31] available through the UCSC genome browser [32]. These results show that rs6137010 is a *cis*-eQTL of *SNRPB* in Asian populations. Changes in the expression level of *SNRNPB* have been reported to affect alternative splicing and abundance of a large number of RNA processing factors [27]. rs6137010 has two alleles, T and C, with C the minor allele in Asian populations but the major allele in the other HapMap3 populations. Asian individuals carrying the C allele at this SNP had higher expression levels of *SNRPB* (Figure 4A) and higher RS-scores (Figure 4B). This suggests that the association between rs6137010 and inter-individual variation in RNA stability could be mediated by changes in *SNRPB* expression levels.

To identify genes across the human genome whose expression levels are significantly associated with rs6137010, we carried out *trans*-eQTL mapping for this SNP by fitting Spearman rank correlation models and considering only associations with FDR<0.1. FDRs were calculated using the Benjamini and Hochberg procedure [33] as implemented in R [34]. We found 6,396 and 2,585 genes associated with rs6137010 in CHB and JPT, respectively. Among these, 3,194 (in CHB) and 429 (in JPT) genes were positively correlated with the minor allele count of rs6137010. Among the genes that were associated with rs6137010, 25.2% were putative targets for AU-rich element decay, compared to 17.6% of other genes (*p* = 0.01, Fisher exact test). We did not find any genes significantly associated with the SNP in other populations using the same FDR threshold. We carried out Gene Ontology (GO) analyses using DAVID [35] for the positively correlated genes and, interestingly, found that they were enriched for the GO term ribonucleoprotein complex in both CHB (*p* = 1.9×10^−25^; Table S5) and JPT (*p* = 3.7×10^−5^). The ribonucleoprotein complex is known to be involved in many steps of RNA processing such as pre-mRNA splicing and RNA transportation and stabilization. Both *HNRNPA2B1* and *SNRPB* mentioned above belong to the ribonucleoprotein complex. These results indicate that rs6137010 is a *trans*-eQTL cluster that is disproportionately associated with the expression levels of ribonucleoprotein complex genes.

**Figure 5:**
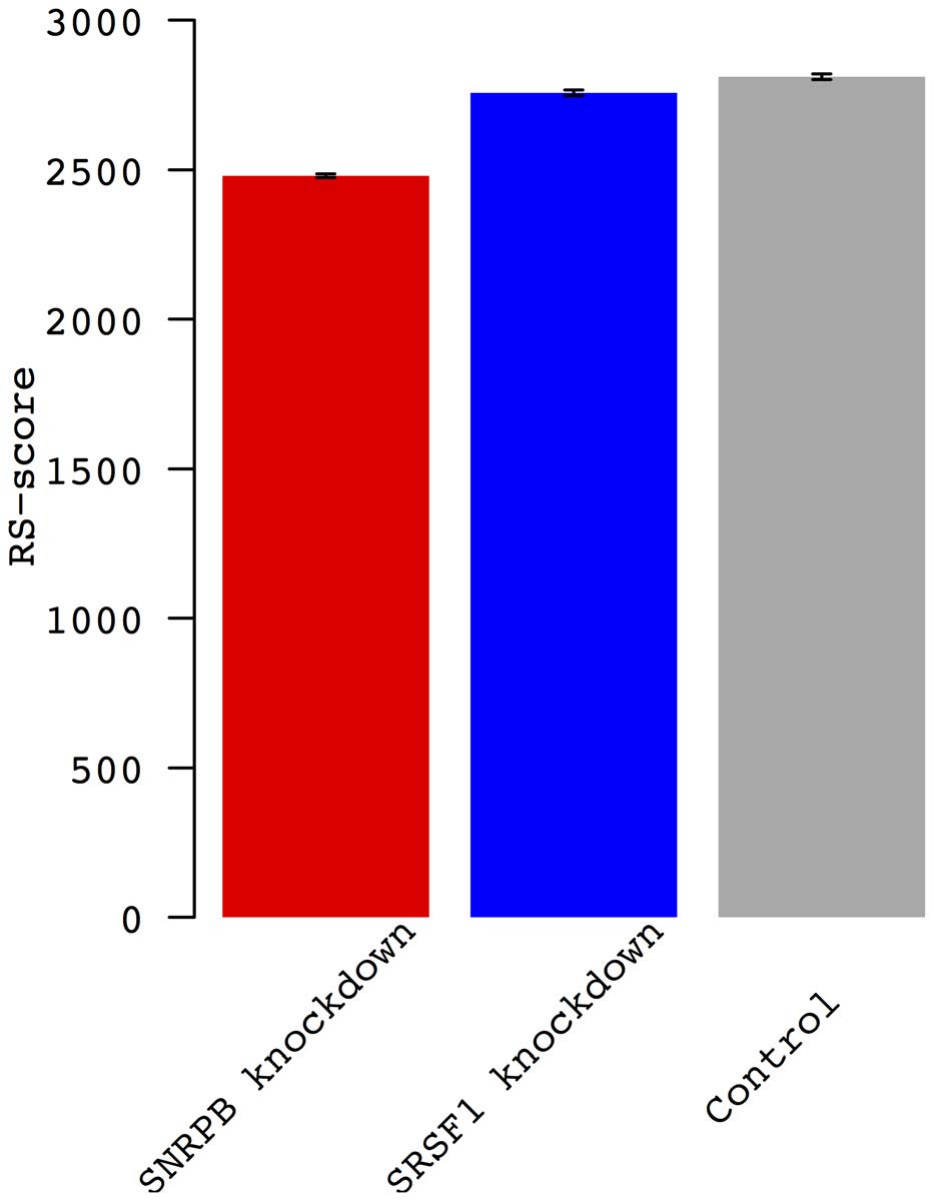
RS-scores calculated from three samples - *SNRPB* knockdown, *SRSF1* knockdown and control. The control corresponds to the sample transfected with nontargeting siRNA. Error bars represent two standard errors.

We next turned to investigating further the possible role of *SNRPB* in mediating the association of rs6137010 with the RS-score. We obtained gene expression microarray data generated from HeLa cells in which *SNRPB* was knocked down and compared to controls [27]. Using the HeLa half-life data [15] we calculated and compared RS-scores between the two conditions and found a significant reduction of the RS-score in *SNRPB* knockdown (*p* = 1.2×10^−6^ from a two-tailed *t* test; Figure 5). This is consistent with expectations because depletion of *SNRPB* reduces the levels of many RNA processing genes [27], potentially affecting the stability of RNA across the transcriptome. Furthermore, the genes that were differentially expressed upon *SNRPB* knockdown were enriched for genes that showed the strongest association (FDR<0.01) with rs6137010 in CHB (*p* = 0.002 from two-tailed Fisher's exact test). These results suggest that rs6137010, by modulating the expression of *SNRPB*, may be directly responsible for inter-individual variation in the RS-score in CHB. Interestingly, the distribution of the RS-score was bi-modal in both CHB and JPT (Figure 4B), consistent with the existence of an associated locus with a large effect size. It is tempting to speculate that an ungenotyped causal SNP in strong linkage disequilibrium with rs6137010 may stratify the samples between the two modes of the distribution. Higher resolution genotype data will be necessary to test this hypothesis.

The RS-score of the knockdown of another splicing factor, *SRSF1*, is also significantly lower than of the control (*p* = 3.4×10^−4^ from a two-tailed *t* test), but significantly higher than of the *SNRPB* knockdown (*p* = 1.1×10^−9^ from a two-tailed *t* test) (Figure 5).This indicates that knocking down *SNRPB* has stronger effect on the RS-score than knocking down *SRSF1*. This is not surprising because *SNRPB* has been found to have a stronger impact than *SRSF1* on the inclusion levels of alternative exons that are enriched for genes encoding RNA processing [27]. *SNRPB*, which plays a central role in modulating expression levels of many RNA processing factors [27], might therefore have the strongest influence in the RS-score among RNA processing factors. Previous studies discovered the involvement of several splicing factors in RNA stability [36], [37]. Thus, the core splicing factor *SNRPB* may have an important role in RNA stability as well.

## Conclusions

Genetic variants that affect RNA stability in *cis* have been shown to contribute to inter-individual variation in gene expression [25]. Here we demonstrate that the effects of knocking down the expression of *HNRNPA2B1* that stabilizes a large number of RNAs can be detected from gene expression data. In particular, the expression of genes expressing transcripts with a long half-life is reduced relative to genes with short half-life transcripts. We defined the RS-score to summarize the relative expression of long-lived compared to short-lived transcripts. Treating the RS-score as a quantitative trait, we performed genome-wide association and identified a locus on chromosome 20p13 that is strongly associated with the RS-score in two Asian populations. This locus is a *cis*-eQTL for *SNRPB*, a core component of the spliceosome that has previously been shown to affect the expression of many RNA processing factors [27]. We propose that the *cis*-eQTL of *SNRPB* may be directly responsible for the association of the RS-score with this locus. Consistent with this model, knockdown of *SNRPB* results in a significant reduction in the RS-score.

## Methods

### Data

Processed gene expression data generated using the Illumina whole genome expression array from 726 lymphoblastoid cell lines (LCLs) in eight HapMap3 populations (CEU, CHB, GIH, JPT, LWK, MEX, MKK, and YRI) by [26] were downloaded from ArrayExpress [38]. Single nucleotide polymorphisms (SNPs) for the same 726 individuals were obtained from HapMap3 (release 2) [39]. SNPs with minor allele frequency (MAF) ≤1% in a population were excluded. This resulted in between 1.1 million and 1.3 million SNPs per population. Half-life data for 11,052 mRNAs and 1,418 ncRNAs in HeLa cells, and for 8,344 genes in B-cells were obtained from Tani *et al*. [15] and Friedel *et al*. [14], respectively.

### RNA stability score

We defined the RNA stability score (RS-score), as a measure of the relative expression levels of long-lived and short-lived transcripts in a sample. We first classified all genes as either expressing long or short lived RNAs, by setting a threshold on an available RNA half-life or decay rate data set. Specifically, for the HeLa half-life data [15], we chose the same threshold used by the authors to determine whether a gene expresses long-lived (half-life ≥4 hours) or short-lived (half-life <4 hours) RNA. For the RNA decay rate data [13], a gene was considered as expressing long-lived RNA if its decay rate was greater than the average across genes (corresponding to a relative decay rate greater than 0) and as short-lived if its decay rate was less than average (corresponding to values less than 0). We then ranked all genes in the sample by their expression levels (a higher expression level corresponds to higher rank value). Finally, the RS-score is defined as the difference in the mean rank of genes expressing long-lived RNAs and genes expressing short-lived RNAs. Therefore, higher RS-scores correspond to higher relative expression of genes with longer half-life, consistent with more efficient stabilization of RNA.

### Genome-wide association test

Assuming an additive mode of inheritance, we performed linear regression analysis to assess association of RS-score with SNP genotypes, using PLINK v1.07 [40]. We included gender as a covariate in the linear model to correct for any sex bias. To combine samples from different populations, we carried out a principal component analysis (PCA) as implemented in the Eigensoft 4.2 [29], [41]. To correct for population stratification in genome-wide association tests, we included the first five principal components in addition to gender as covariates in the linear models.

### Permutation testing

Applying a permutation testing procedure by Hirschhorn and Daly [42], in each GWAS test, we carried out 1000 permutations. In each permutation, we randomly shuffled the phenotype values, re-ran the GWAS and recorded the best (lowest) p-value from each run. Finally, we counted how many of these 1000 lowest p-values are less than or equal to the original p-value being evaluated. The permutation *p* is defined as this number divided by 1000 (i.e. the proportion of the 1000 lowest p-values that are less than or equal to the original p-value).

### Analysis of RNA-seq data from *SNRPB* knockdown samples

We downloaded RNA-seq data generated by Saltzman *et al*. [27] from samples in which *SNRPB* or *SRSF1* were knocked down as well as control samples. The data consisted of three samples for each knock down and three control samples. We mapped the RNA-seq reads to the human genome, build hg19, using Tophat 1.4.1 (with default parameters) [43] and estimated expression levels of RefSeq genes using Cufflinks 1.3.0 (with default parameters) [44]. Using the HeLa half-life data [15], we calculated the RS-score for each of the three samples.

## Supporting Information

Figure S1 **Gene expression levels in HNRNPA2B1 knockdown relative to control are shown separately for genes expressing short-lived (golden) and long-lived (dark green) RNAs in three independent replicates (Rep1, Rep2, and Rep3).** P-values are from Wilcoxon rank sum tests that were used to compare expression levels between these two groups of genes.
(TIFF)

Figure S2 **First principal component (PC1) versus second principal component (PC2) for all 726 individuals from 8 populations.**
(TIFF)

Figure S3 **Manhattan plots for GWA with RS-score in different populations and combined populations.** Each Manhattan plot shows the distribution of -log10 of the P-values from tests of association between individual SNP markers and the RS-score.
(TIFF)

Figure S4 **P-P plots of the association with RS-score. The expected (X-axis) shows -log10 of random values, drawn from the uniform distribution.** The observed (Y-axis) shows -log10 of the P-values from tests of association between individual SNP markers and the RS-score. The red line is used to compare the expected and observed values.
(TIFF)

Table S1 **Summary of samples in the eight Hapmap3 populations.**
(DOCX)

Table S2 **Spearman correlation between HNRNPA2B1 and the RS-score.**
(DOCX)

Table S3 **Association between cis-eQTL of HNRNPA2B1 and the RS-score.**
(DOCX)

Table S4 **Genomic inflation factors (lambda) in different populations.**
(DOCX)

Table S5 **Top GO terms for genes positively correlated with rs6137010 in CHB.**
(DOCX)
