# Supplementary figures and images for: Integrative Analysis of mRNA Expression and Half-Life Data Reveals Trans-Acting Genetic Variants Associated with Increased Expression of Stable Transcripts
Source: PLoS One. 2013 Nov 18;8(11):e79627. doi: 10.1371/journal.pone.0079627 (PMC3832542; doi:10.1371/journal.pone.0079627)

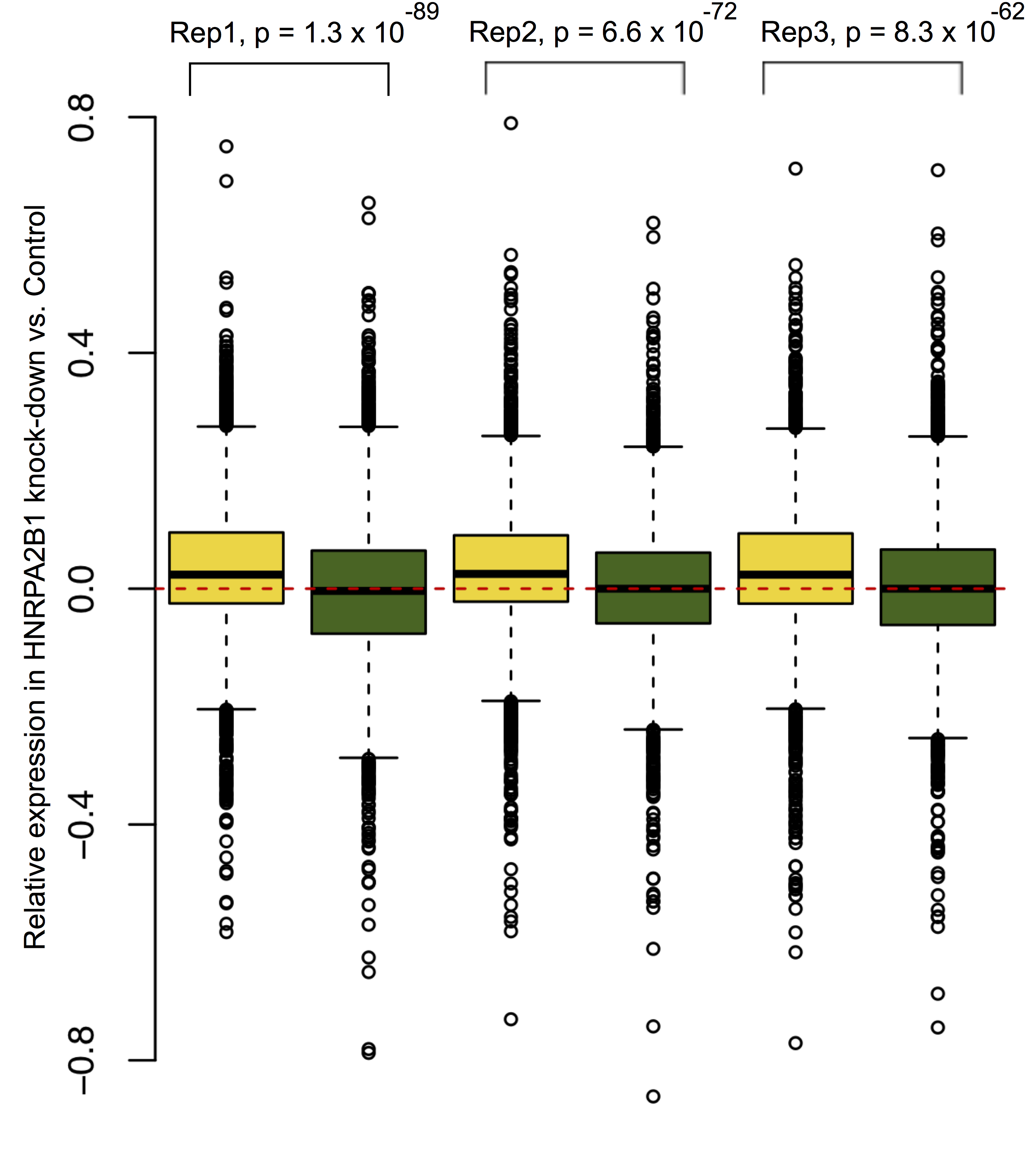

Supplement: Figure S1 — Gene expression levels in HNRNPA2B1 knockdown relative to control are shown separately for genes expressing short-lived (golden) and long-lived (dark green) RNAs in three independent replicates (Rep1, Rep2, and Rep3). P-values are from Wilcoxon rank sum tests that were used to compare expression levels between these two groups of genes. (TIFF) [file pone.0079627.s001.tiff]

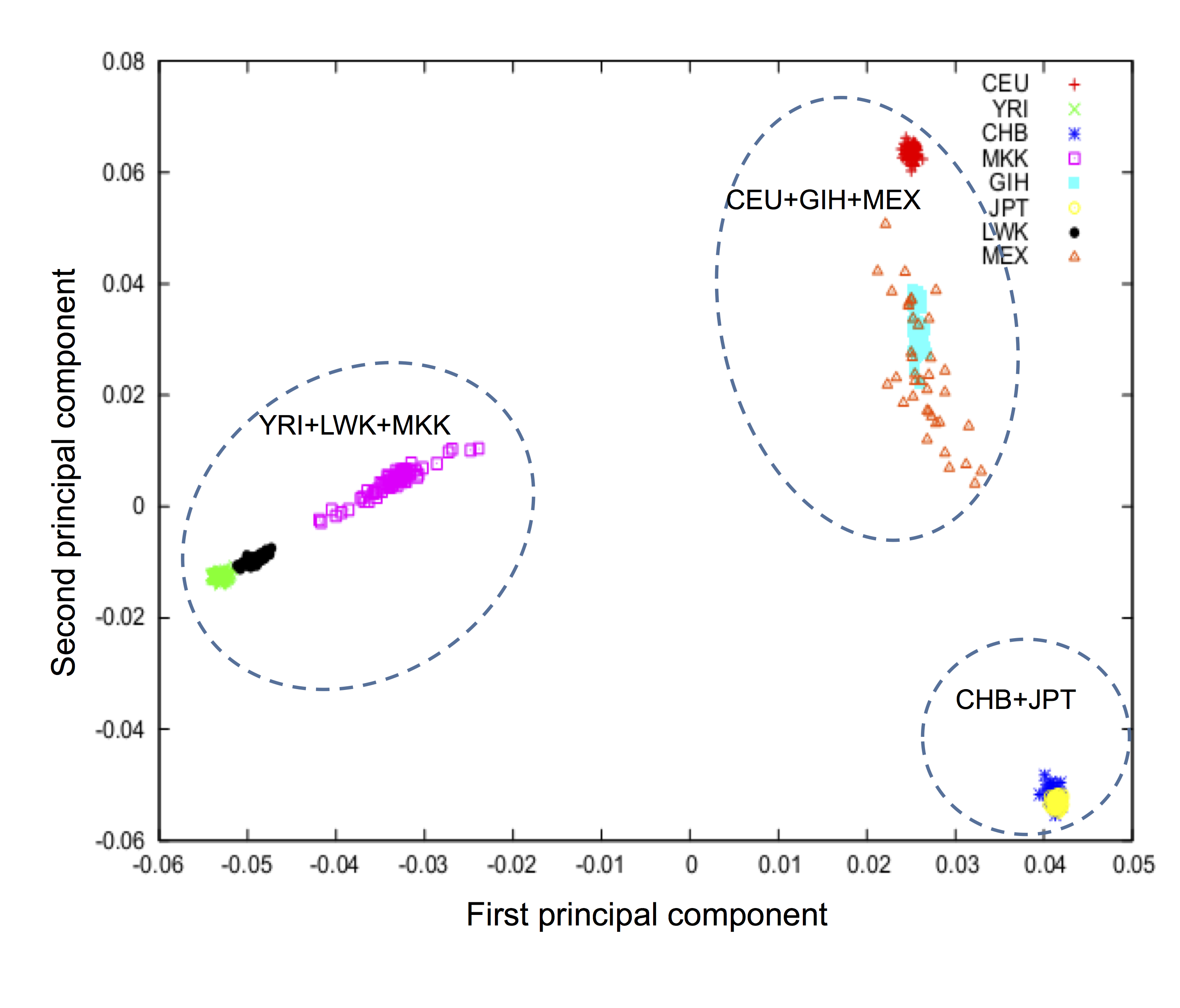

Supplement: Figure S2 — First principal component (PC1) versus second principal component (PC2) for all 726 individuals from 8 populations. (TIFF) [file pone.0079627.s002.tiff]

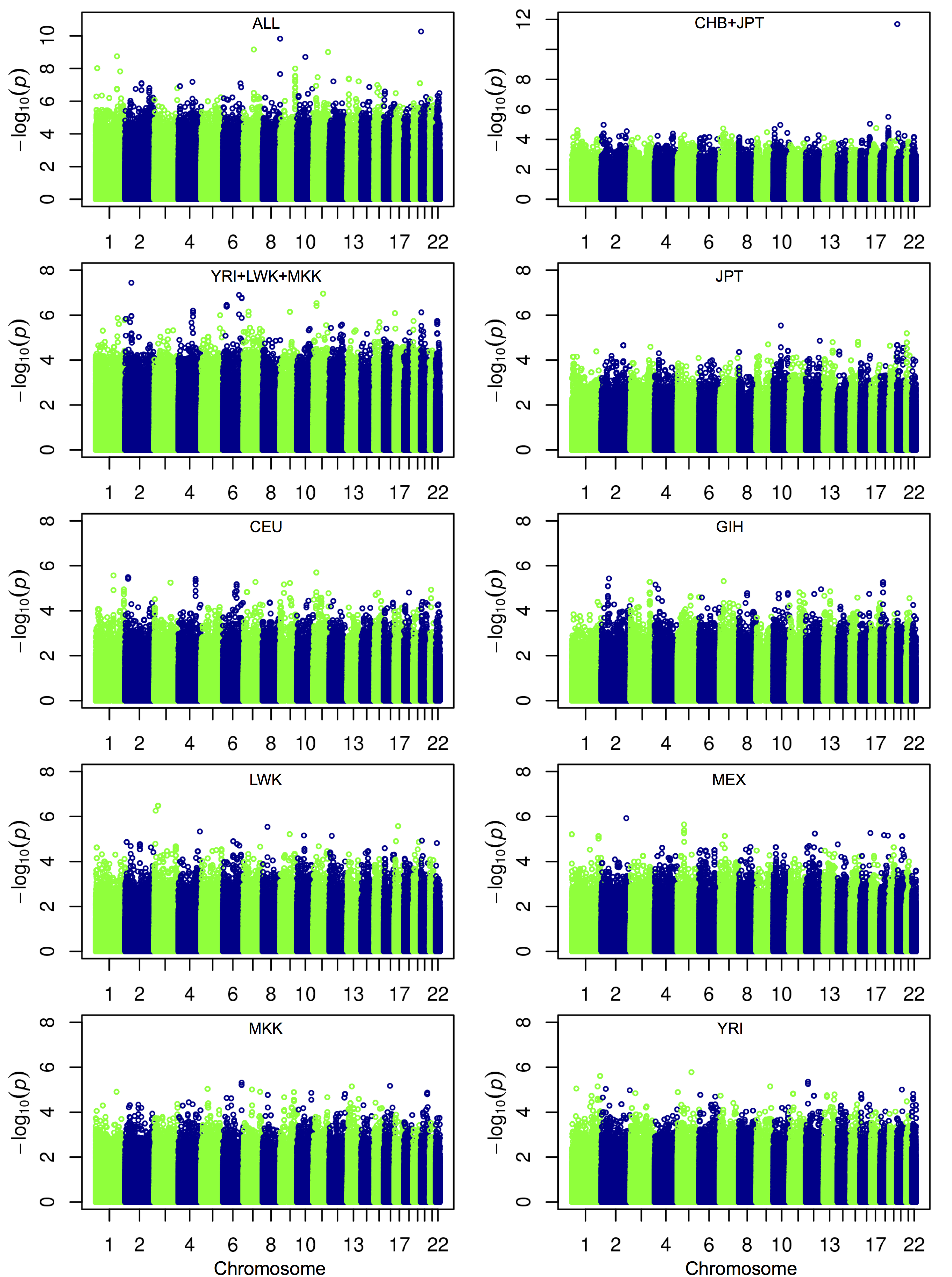

Supplement: Figure S3 — Manhattan plots for GWA with RS-score in different populations and combined populations. Each Manhattan plot shows the distribution of -log10 of the P-values from tests of association between individual SNP markers and the RS-score. (TIFF) [file pone.0079627.s003.tiff]

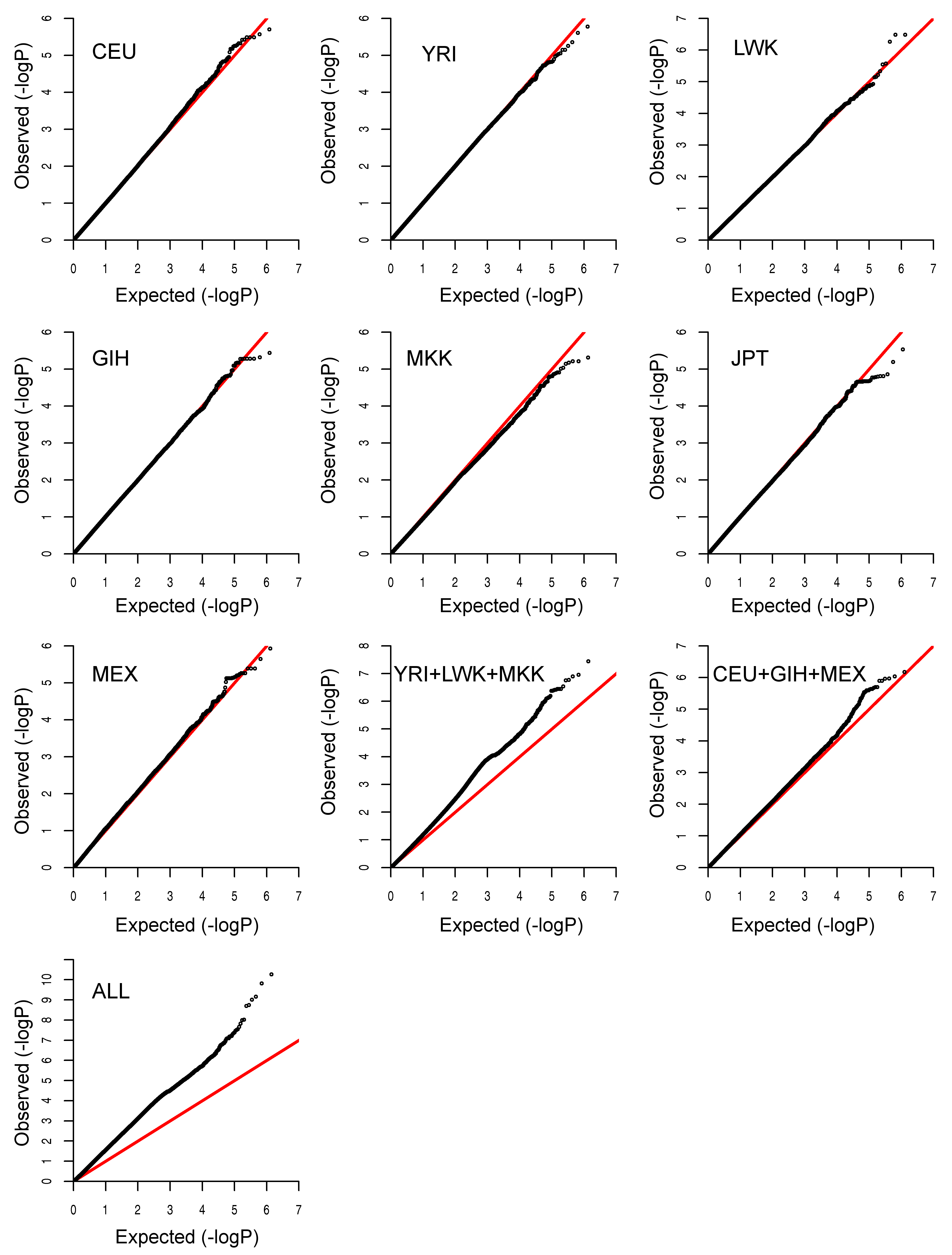

Supplement: Figure S4 — P-P plots of the association with RS-score. The expected (X-axis) shows -log10 of random values, drawn from the uniform distribution. The observed (Y-axis) shows -log10 of the P-values from tests of association between individual SNP markers and the RS-score. The red line is used to compare the expected and observed values. (TIFF) [file pone.0079627.s004.tif]
