# Supplementary material for: Integrative Analysis of mRNA Expression and Half-Life Data Reveals Trans-Acting Genetic Variants Associated with Increased Expression of Stable Transcripts
Source: PLoS One. 2013 Nov 18;8(11):e79627. doi: 10.1371/journal.pone.0079627 (PMC3832542; doi:10.1371/journal.pone.0079627)
Supplement: Table S1 — Summary of samples in the eight Hapmap3 populations. (DOCX) [file pone.0079627.s005.docx]

Table S1. Summary of samples in the eight Hapmap3 populations

| **Population** | **Population detail** | **Number of samples** |
| --- | --- | --- |
| CEU | Caucasians living in Utah USA,  of northern and western European ancestry | 109 |
| CHB | Han Chinese from Beijing, China | 80 |
| GIH | Gujarati Indians in Houston, TX, USA | 82 |
| JPT | Japanese in Tokyo, Japan | 82 |
| LWK | Luhya in Webuye, Kenya | 82 |
| MEX | Mexican ancestry in Los Angeles, CA, USA | 45 |
| MKK | Maasai in Kinyawa, Kenya | 138 |
| YRI | Yoruba in Ibadan, Nigeria | 108 |
