# Supplementary material for: Integrative Analysis of mRNA Expression and Half-Life Data Reveals Trans-Acting Genetic Variants Associated with Increased Expression of Stable Transcripts
Source: PLoS One. 2013 Nov 18;8(11):e79627. doi: 10.1371/journal.pone.0079627 (PMC3832542; doi:10.1371/journal.pone.0079627)
Supplement: Table S2 — Spearman correlation between HNRNPA2B1 and the RS-score. (DOCX) [file pone.0079627.s006.docx]

Table S2. Spearman correlation between *HNRNPA2B1* and the RS-score

| **Population** | **Rho** | **P-value** |
| --- | --- | --- |
| YRI | 0.38 | 5.0 x 10^-05^ |
| CHB | 0.48 | 8.4 x 10^-06^ |
| MKK | 0.18 | 3.3 x 10^-02^ |
| GIH | 0.30 | 6.1 x 10^-03^ |
| JPT | 0.28 | 1.2 x 10^-02^ |
| LWK | 0.30 | 5.7 x 10^-03^ |
| MEX | 0.33 | 2.7 x 10^-02^ |
| CEU | 0.06 | 5.5 x 10^-01^ |
