# Supplementary material for: Integrative Analysis of mRNA Expression and Half-Life Data Reveals Trans-Acting Genetic Variants Associated with Increased Expression of Stable Transcripts
Source: PLoS One. 2013 Nov 18;8(11):e79627. doi: 10.1371/journal.pone.0079627 (PMC3832542; doi:10.1371/journal.pone.0079627)
Supplement: Table S3 — Association between cis-eQTL of HNRNPA2B1 and the RS-score. (DOCX) [file pone.0079627.s007.docx]

Table S3. Association between *cis*-eQTL of *HNRNPA2B1* and the RS-score

| **Population** | **SNP** | **P1** | **P2** | **P2_BH** |
| --- | --- | --- | --- | --- |
| CHB | rs17153827 | 4.5 x 10^-05^ | 2.2 x 10^-03^ | 1.6 x 10^-02^ |
| CHB | rs17154015 | 6.5 x 10^-04^ | 5.0 x 10^-03^ | 2.0 x 10^-02^ |
| LWK | rs10242687 | 1.9 x 10^-04^ | 2.7 x 10^-03^ | 1.6 x 10^-02^ |
| LWK | rs1125542 | 3.6 x 10^-05^ | 8.4 x 10^-03^ | 2.5 x 10^-02^ |

The second column shows four *cis*-eQTLs for *HNRNPA2B1* that are significantly associated with RS-score. *cis*-eQTL mapping for *HNRNPA2B1* was carried out, following a pipeline from Stranger *et al.* [[1](#_ENREF_1)], by performing Spearman correlation tests between SNPs located within 500 Kb of the transcription start site of *HNRNPA2B1* and its expression level. Only SNPs corresponding to tests having P-values ≤ 0.001 are considered as *cis*-eQTLs, and these P-values are shown in the third column (P1). The fourth column (P2) contains the association P-values (only those values ≤ 0.01 are shown) between these *cis*-eQTLs and the RS-score. The final column (P2_BH) is the P-values of P2, corrected for multiple testing using the Benjamini and Hochberg procedure.

# References

1. Stranger BE, Montgomery SB, Dimas AS, Parts L, Stegle O, et al. (2012) Patterns of cis regulatory variation in diverse human populations. PLoS Genet 8: e1002639.
