# Supplementary material for: Integrative Analysis of mRNA Expression and Half-Life Data Reveals Trans-Acting Genetic Variants Associated with Increased Expression of Stable Transcripts
Source: PLoS One. 2013 Nov 18;8(11):e79627. doi: 10.1371/journal.pone.0079627 (PMC3832542; doi:10.1371/journal.pone.0079627)
Supplement: Table S4 — Genomic inflation factors (lambda) in different populations. (DOCX) [file pone.0079627.s008.docx]

Table S4. Genomic inflation factors (lambda) in different populations.

| **Population** | **Lambda** |
| --- | --- |
| YRI | 1.005 |
| CHB | 1.017 |
| MKK | 1.000 |
| GIH | 1.024 |
| JPT | 1.014 |
| LWK | 1.000 |
| MEX | 1.060 |
| CEU | 1.011 |
| CHB+JPT | 1.020 |
| CEU+GIH+MEX | 1.086 |
| YRI+LWK+MKK | 1.262 |
| ALL | 1.900 |
