# Supplementary material for: Integrative Analysis of mRNA Expression and Half-Life Data Reveals Trans-Acting Genetic Variants Associated with Increased Expression of Stable Transcripts
Source: PLoS One. 2013 Nov 18;8(11):e79627. doi: 10.1371/journal.pone.0079627 (PMC3832542; doi:10.1371/journal.pone.0079627)
Supplement: Table S5 — Top GO terms for genes positively correlated with rs6137010 in CHB. (DOCX) [file pone.0079627.s009.docx]

Table S5. Top GO terms for genes positively correlated with rs6137010 in CHB

| **Term** | **P-value** | **Bonferroni** |
| --- | --- | --- |
| membrane-enclosed lumen | 8.3 x 10^-39^ | 5.9 x 10^-36^ |
| intracellular organelle lumen | 1.0 x 10^-38^ | 7.3 x 10^-36^ |
| organelle lumen | 4.2 x 10^-36^ | 3.0 x 10^-33^ |
| Mitochondrion | 2.3 x 10^-35^ | 1.6 x 10^-32^ |
| nuclear lumen | 7.2 x 10^-30^ | 5.1 x 10^-27^ |
| mitotic cell cycle | 9.9 x 10^-28^ | 4.3 x 10^-24^ |
| cell cycle | 2.8 x 10^-26^ | 1.2 x 10^-22^ |
| mitochondrial part | 5.8 x 10^-26^ | 4.1 x 10^-23^ |
| ribonucleoprotein complex | 1.9 x 10^-25^ | 1.3 x 10^-22^ |
| Nucleoplasm | 2.2 x 10^-25^ | 1.6 x 10^-22^ |
| cell cycle process | 7.6 x 10^-24^ | 3.3 x 10^-20^ |
| organelle envelope | 2.8 x 10^-23^ | 2.0 x 10^-20^ |
| Envelope | 5.1 x 10^-23^ | 3.6 x 10^-20^ |
| RNA processing | 6.6 x 10^-20^ | 2.8 x 10^-16^ |
